# How Cooking Time Affects In Vitro Starch and Protein Digestibility of Whole Cooked Lentil Seeds versus Isolated Cotyledon Cells

**DOI:** 10.3390/foods12030525

**Published:** 2023-01-24

**Authors:** Dorine Duijsens, Sarah H. E. Verkempinck, Audrey De Coster, Katharina Pälchen, Marc Hendrickx, Tara Grauwet

**Affiliations:** Laboratory of Food Technology, Leuven Food Science and Nutrition Research Centre (LfoRCe), Department of Microbial and Molecular Systems (M2S), KU Leuven, Kasteelpark Arenberg 22, PB 2457, 3001 Leuven, Belgium

**Keywords:** lentil, thermal treatment, amylolysis, proteolysis, INFOGEST, digestion kinetics, individual cotyledon cell

## Abstract

Lentils are sustainable sources of bioencapsulated macronutrients, meaning physical barriers hinder the permeation of digestive enzymes into cotyledon cells, slowing down macronutrient digestion. While lentils are typically consumed as cooked seeds, insights into the effect of cooking time on microstructural and related digestive properties are lacking. Therefore, the effect of cooking time (15, 30, or 60 min) on in vitro amylolysis and proteolysis kinetics of lentil seeds (CL) and an important microstructural fraction, i.e., cotyledon cells isolated thereof (ICC), were studied. For ICC, cooking time had no significant effect on amylolysis kinetics, while small but significant differences in proteolysis were observed (*p* < 0.05). In contrast, cooking time importantly affected the microstructure obtained upon the mechanical disintegration of whole lentils, resulting in significantly different digestion kinetics. Upon long cooking times (60 min), digestion kinetics approached those of ICC since mechanical disintegration yielded a high fraction of individual cotyledon cells (67 g/100 g dry matter). However, cooked lentils with a short cooking time (15 min) showed significantly slower amylolysis with a lower final extent (~30%), due to the presence of more cell clusters upon disintegration. In conclusion, cooking time can be used to obtain distinct microstructures and digestive functionalities with perspectives for household and industrial preparation.

## 1. Introduction

Pulses can be an important part of healthy and sustainable diets (e.g., the Mediterranean diet), as they are a source of (slowly digestible) starch, protein, minerals, and (soluble and insoluble) fiber [1]. Before consumption, pulses require processing to reach palatability. During cooking, starch gelatinizes, protein denatures, and the gradual solubilization of pectin leads to weakening of the cell middle lamella and softening of the seeds [2,3,4]. As a result, the mechanical disintegration (e.g., mastication) of cooked pulses causes cotyledon cells to remain intact and separate rather than break [3,4]. In pulse cells, macronutrients such as starch and protein are encapsulated within the cell wall. Both the cell wall and the cytoplasmic protein matrix have been widely reported to form a barrier, slowing and hindering macronutrient (starch and protein) digestion [5]. Retarded nutrient digestibility in intact cells has, in turn, been related to in vivo benefits, among which are increased satiety [6,7] and a low glycemic index [8,9], which is, in turn, correlated to a reduction in the risk of modern lifestyle diseases [10].

While typical hurdles for pulse consumption are their inconvenient preparation and typical aroma [11], the rising interest in lentils could be related to their shorter cooking time [12] and more pleasant aroma compared to other pulses [13,14]. Lentils (*Lens culinaris*) are lens-shaped pulses, with a high protein (20.6–29.2%) and fiber (6.8–14.7%) but low lipid (0.8–2.2%) content compared to other pulse types [15]. Recently, lentil ingredients are being increasingly incorporated into several innovative food products, such as crackers, cookies, and pasta [16,17]. While the properties and application of lentil-based food ingredients (with or without cellular intactness) are being studied [18,19], pulses are still mostly consumed in the form of cooked seeds. Therefore, both at the level of industrial and household preparation, it is important to obtain insight into the effect of cooking on the nutrient digestibility of lentils. 

The influence of cooking time on the microstructural and linked in vitro macronutrient digestibility of several pulse types has been studied, revealing important intrinsic differences between pulse types [15], as elaborated below. Most commonly, since individual cotyledon cells can be considered a representative fraction of disintegrated cooked pulse seeds, isolated cotyledon cells (ICC) are considered [20,21,22,23,24,25,26,27,28,29,30]. Since this approach entails the omission of certain fractions (such as cell clusters, fiber-rich seed coat material, and ruptured cells), it can be considered a reductionist approach [30,31]. The study of ICC fractions revealed a positive correlation between the applied cooking time and the starch and protein digestion rate and extent of isolated of black beans, common beans (*Phaseolus vulgaris*), and Bambara groundnut (*Vigna subterranea*) [31,32,33]. In contrast, cotyledon cells isolated from, e.g., peas (*Pisum sativum*) and chickpeas (*Cicer arietinum*), did not show an effect of cooking time on the overall macronutrient digestibility [31]. In the context of ICC, pulse type-specific differences in digestibility have been linked to, among others, the starch-to-protein ratio and cell wall properties (such as thickness, porosity, and composition) [5,31,34]. Recently, next to ICC, the effect of cooking time on the microstructural and (resulting) digestion properties of whole seeds are being studied as well. Consistently, an increased fraction (or yield) of individual cells upon mechanical disintegration was reported with increasing seed cooking times [21,32]. For several pulse types (e.g., peas and chickpeas), studies reported increased amylolysis and proteolysis rates and extents with increasing seed cooking times due to the separation of cell clusters [31,35]. In contrast, another study reported a decrease in the macronutrient digestion rate with increasing cooking times due to decreased cell breakage for Bambara groundnuts [36]. 

The abovementioned studies indicated that the cooking time can be an important parameter affecting nutrient digestibility in pulses, with important differences between pulse types. However, insight into the effect of cooking time on the microstructural and linked digestive properties of well-accepted lentils is lacking. Therefore, three different processing intensities (cooking times) were selected at which lentil seeds were palatable and starch gelatinization was complete, but the seeds showed differences in particle size distributions upon mechanical disintegration. Starch and protein digestion kinetics were determined for both the whole cooked seed material and cotyledon cells isolated thereof and compared and linked to microstructural properties. The use of the INFOGEST consensus static in vitro protocol [37] ensures comparability of the data with other works on pulse digestibility.

## 2. Materials and Methods

### 2.1. Materials

Green lentils (Dupuy type) from Canada (harvested in August 2019) were donated by Casibeans (Melsele, Belgium). After sorting, the raw seeds (<10% moisture) were stored at −40 °C. Storage below the glass transition temperature prevents biochemical changes (such as the development of the hard-to-cook phenomenon), ensuring stability of the seeds until usage [38,39]. The Total Starch Kit was obtained from Megazyme (Bray, Ireland). KCl, MgCl_2_(H_2_O)_6_, NaOH, and sodium potassium tartrate were purchased from Acros Organics (Geel, Belgium). NaHCO_3_, NaCl, and KH_2_PO_4_ were obtained from VWR (Leuven, Belgium). All other chemical reagents and enzymes were purchased from Merck KgaA (Darmstadt, Germany).

### 2.2. Thermal Processing of Lentil Seeds and Isolation of Cotyledon Cells

**Figure 1:**
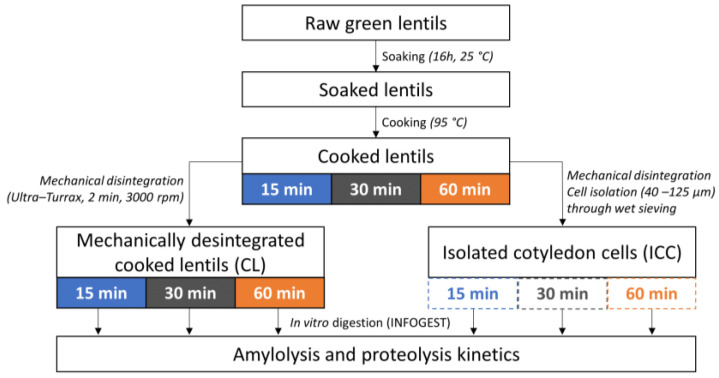
Experimental approach applied to study the effect of cooking time on in vitro starch and protein digestion kinetics in cooked lentil seeds and cotyledon cells isolated thereof.

The applied experimental approach is shown in Figure 1. Whole cooked lentils (CL) and isolated cotyledon cells (ICC) were produced following a procedure based on Pallares Pallares et al. (2018) [21]. Upon preparation, the material was frozen in liquid nitrogen and stored at −40 °C until sample characterization and simulated digestion. As reported earlier, no effect of snap freezing and frozen storage (−40 °C) on the microstructural and digestive properties could be observed [29,32,33].

The cooking times were selected based on preliminary experiments and the literature, with cooking times from 8 to 43 min reported for presoaked lentil seeds [40,41,42]. Preliminary experiments showed that presoaked lentils had an acceptable (palatable) texture upon cooking for 15, 30, or 60 min. Moreover, the Differential Scanning Calorimetry (DSC) analysis (see Section 2.3.1, data in Figure S1) indicated complete starch gelatinization for these thermal treatment times. It was hypothesized that the application of these increasing cooking times within the palatable range would lead to increasing degrees of (middle lamella) pectin solubilization and thereby (i) differences in the microstructure (PSD) upon mechanical disintegration of whole cooked seeds and (ii) isolated cotyledon cell fractions with possibly different barrier properties, both potentially affecting macronutrient digestion kinetics [32,33]. 

#### 2.2.1. Whole Cooked Lentil Seeds (CL)

Raw lentils were soaked in demineralized water (1:10 *w*/*v*, 16h, 25 °C), whereafter the soaking water was discarded. To study the impact of cooking time, soaked lentils were independently cooked in demineralized water (1:10 *w*/*v*, 95 °C) for each selected cooking time (15, 30, or 60 min). Upon reaching the required cooking time, samples were cooled to room temperature on ice, and the cooking water was discarded. Cooked lentils were rinsed with demineralized water and drained. Solid foods such as lentils require mechanical disintegration before digestion, either by mastication or processing. Therefore, the cooked lentils were mixed into a slurry with a Simulated Salivary Fluid (SSF, 1.25:1 *m/v*) wet basis under standardized conditions (cfr. Section 2.4.1).

#### 2.2.2. Isolated Cotyledon Cells (ICC)

Lentils cooked for 15, 30, or 60 min (see above) were mixed with demineralized water (1:1 *w*/*v*) and disintegrated (2 min, 3000 rpm) (IKA^®^ T25 Ultra-Turrax, Janke and Kunkel, Germany). Wet sieving by means of a vibratory sieve system (AS200, Retsch, Germany) was performed to isolate cotyledon cells from the slurry (amplitude 2.5 mm, 4 min). The fractions between 40 and 125 µm were composed of individual cotyledon cells, as confirmed microscopically and by laser diffraction (Section 3.1.1). The cellular yield was then calculated on a dry weight basis. 

### 2.3. Sample Characterization

#### 2.3.1. Compositional Analysis

The moisture content of both CL and ICC samples was determined in duplicate as the difference in weight before and after oven drying. For compositional analysis, a portion of all CL and ICC samples were freeze-dried (Alpha 1–4, LSCplus, Martin Christ, Germany) and pounded into a ball mill (MM400, Retsch, Haan, Germany), completely disrupting cellular structures. The starch content was analyzed in duplicate employing a Total Starch Kit (AA/AMG, Megazyme Inc. Bray, Ireland). The nitrogen content was determined by an automated Dumas analysis (CHNS-O Elemental Analyzer, CE instrument, Thermo Fischer Scientific, Waltham, MA, USA) in duplicate. Then, the crude protein content was calculated employing a conversion factor of 5.4 for legumes [43]. Differential Scanning Calorimetry (DSC) (TA instruments, Q2000, New Castle, DE, USA) was performed to confirm complete starch gelatinization of all samples in view of the analysis of starch digestibility, applying the procedure suggested by Salgado-Cruz, Ramírez-Miranda, Díaz-Ramírez, Alamilla-Beltran, & Calderón-Domínguez (2017) [44] with minor modifications reported by Noordraven, Bernaerts, Mommens, Hendrickx, & Van Loey (2021) [45]. Thermal transitions were identified through analysis of the obtained thermograms using TA Universal Analysis software (Version 4.5A, TA Instruments-Waters LLC, New Castle, DE, USA). DSC data are included in Supplementary Materials Figure S1. 

#### 2.3.2. Microscopy

Representative micrographs were used to qualitatively visualize the disappearance of starch and proteins in the digestion pellets (i.e., non-bioaccessible, insoluble fraction obtained upon centrifugation of digests, cfr. Section 2.4.3) as a result of digestion. To visualize starch, a 5% (*w*/*v*) Lugol’s iodine solution was used. Micrographs were taken using an Olympus BX-51 light microscope (Olympus, Optical Co. Ltd., Tokyo, Japan) with cellSense Standard^®^ software employing a 40× magnification objective. Due to the presence of aromatic amino acids, pulse proteins are intrinsically fluorescent [32,46]. Therefore, protein digestion could be visualized under fluorescent light (excitation filter 460–490 nm) using epifluorescence illumination equipment (X-Cite^®^ 120Q, X-Cite^®^ Fluorescence Illumination, EXFO Europe, Hants, UK), as reported earlier [47]. 

#### 2.3.3. Particle Size Distribution

The particle size distribution (PSD) was analyzed using a particle size analyzer employing a Universal Liquid Module (Beckman Coulter, LS 13 320, Miami, FL, USA) in duplicate for each sample, as performed by Noordraven et al. (2021) [45]. Briefly, mechanically disintegrated CL and ICC samples were diluted in demineralized water and injected into the stirred tank (reaching 8–10% obscuration). As the sample was pumped through the measuring cell (rate 30%), dispersed particles scattered the laser light (main illumination source wavelength: 750 nm and halogen light for polarization intensity differential scattering wavelength: 450 nm, 600 nm, and 900 nm). Finally, volumetric PSDs were calculated applying the Fraunhofer optical model.

### 2.4. In Vitro Digestion: INFOGEST 2.0

The digestion of produced lentil samples was simulated in vitro using the static INFOGEST protocol [37]. The activities and concentrations of the used enzymes and bile salts were analyzed prior to digestion, following the procedures proposed by Brodkorb et al. (2019) [37]. Independent digestion tubes were prepared for each analyzed digestion time (5 time points for the gastric and 13 for the small intestinal phase).

#### 2.4.1. Simulated Oral Phase

According to the standardized INFOGEST protocol [37], in each digestion tube, 1.25 g of sample (ICC or cooked lentil seeds) were combined with Simulated Salivary Fluid (SSF, pH 7, 1 mL), CaCl_2_ (0.0063 mL, 0.3 M), and demineralized water (0.125 mL). 

**Figure 2:**
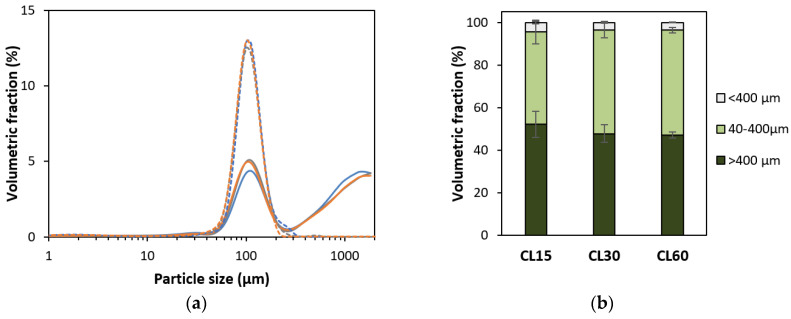
(**a**) Particle size distributions of cooked lentils (CL) and isolated cotyledon cells (ICC) cooked for 15 (**−**), 30 (**−**), or 60 (**−**) minutes. For each sample type, an averaged curve is shown. Full lines represent mechanically disintegrated cooked lentils, and dotted lines represent isolated cotyledon cells. (**b**) Volumetric distribution of CL with a cooking time of 15, 30, or 60 min in different size fractions calculated from duplicate PSD measurements.

Isolated cotyledon cells were used as such, but whole lentil seeds (solid food) required disintegration before simulated digestion. Therefore, cooked lentil seeds were mixed with SSF (1.25:1 *m/v*, Ultra-Turrax, 2 min, 3000 rpm), since this method allows standardization of the disintegration step. Moreover, comparison of PSD data (Section 3.1.1, Figure 2) with the literature data on in vivo masticated bean boluses indicated that this disintegration step yielded a similar microstructural distribution [48].

Previous publications by our research unit [29,32] showed that, in the case of a static in vitro digestion experiment (INFOGEST protocol), salivary amylase had a negligible effect on the amylolysis kinetics due to its short incubation time (2 min) and immediate inactivation upon initiating the gastric phase at pH 3 [49]. Therefore, since the aim of this work was to compare macronutrient digestion trends and rates for differently treated lentil samples, salivary amylase was not incorporated in the simulated oral phase [29,32]. 

#### 2.4.2. Simulated Gastric Phase

The simulated metabolites were mixed with simulated gastric fluid (SGF, pH 3, 2 mL) and CaCl_2_ (0.0013 mL, 0.3 M). The pH was adapted to 3 (2 M HCl). Demineralized water and a porcine pepsin solution were added to obtain a final volume of 5 mL and a pepsin activity of 2000 U/mL. Digestion tubes were incubated under end-over-end rotation (37 °C, 120 min, 70 rpm). At 5 predetermined gastric digestion times, independent samples were taken, and the enzymatic activity was stopped thermally (5 min, 98 °C).

#### 2.4.3. Simulated Small Intestinal Phase

Simulated intestinal fluid (SIF, pH 7, 2.125 mL), CaCl_2_ (0.3 M, 0.01 mL), and fresh bile solution (160 mM in SIF, 0.625 mL) were added to the chyme. The pH was brought to 7 (1 M NaOH). Then, demineralized water and pancreatic enzyme solution (in SIF) were added, finally reaching a volume of 10 mL; a bile concentration of 10 mM; and α-amylase, trypsin, and chymotrypsin activities of, respectively, 200, 100, and 25 U/mL. Thirteen individual digestion tubes were incubated (180 min, 37 °C). At 13 predetermined times, independent samples were taken, followed by enzyme inactivation. 

For the gastric and small intestinal phases, enzyme blanks (containing no sample but all simulated fluids and enzymes) were prepared. All thermally inactivated digestion tubes were centrifuged (5 min, 2000× *g*, 25 °C) (Sigma 4–16 KS centrifuge, Sigma, Osterode am Harz, Germany), after which the supernatant and insoluble, non-bioaccessible pellet were separated, snap-frozen, and stored (−40 °C) until analysis. 

### 2.5. Quantitative Evaluation of Macronutrient Digestion 

#### 2.5.1. Determination of Digested Starch 

The extent of starch digestion (%) was evaluated using the dinitrosalicylic (DNS) method [50,51]. Briefly, 2 mL of (diluted) supernatant was mixed with 1 mL DNS in duplicate for each digestion time. After incubation (15 min, 100 °C), samples were cooled down, 9 mL Milli-Q was added, and the absorbance was read at 540 nm. For each sample, the reducing sugar concentration was calculated in terms of maltose equivalents using a calibration curve (0.5–2.0 mg/mL maltose) and converted into starch (conversion factor 0.95, Equation (1)). Since no salivary amylase was incorporated into the static in vitro digestion protocol, starch digestion was quantified as a function of small intestinal digestion time only.

(1) $$Digested\;starch\;\left(\%\right)=\frac{maltose\;equivalents\times 0.95}{total\;starch\;content}\times 100$$

#### 2.5.2. Determination of Readily Bioaccessible Protein

The course of proteolysis was evaluated as a function of digestion time by applying the spectrophotometric o-phthaldialdehyde (OPA) method [52,53]. As widely applied, the *readily bioaccessible* fraction (*NH_2TCA_*) was quantified by measuring α-amino groups released as a function of the digestion time [29,32,54,55]. Large peptides were precipitated from thermally inactivated supernatants through the addition of 3.2% TCA. Then, samples were centrifuged (10,000× *g*, 25 °C, 30 min) (Microfuge 22R, Beckman Coulter Inc., Indianapolis, IN, USA) and the obtained supernatants filtered (0.25 µm pore size). The resulting fraction is considered to contain small peptides and amino acids that are readily absorbable (as such) at the brush border [56]. After the digested samples, enzyme blanks (no samples but all reagents, *NH_2initial_*) were analyzed as well. The readily bioaccessible peptide fraction at each digestion time was expressed as a proportion (%) of the *total amount of α-amino groups in the (undigested) sample* (*NH*_2*total*_). This fraction was obtained by adding 1 mL 6 N HCl to 5 mg of sample and hydrolyzing in duplicate for 24 h at 110 °C. After removing the acid by rotary evaporation (Heidolph Instruments, Schwabach, Germany), the remaining fraction was redissolved in 5 mL Milli-Q water and filtered. 

Fresh OPA reagent was prepared for each set of analyses, as elaborated by Zahir et al. (2018) [53]. Briefly, 0.4 mL of diluted sample and 3 mL of OPA reagent were mixed, and after 2 min incubation in the dark, the absorbance was read at 340 nm (in duplicate). The detected α-amino groups were converted to L-serine equivalents employing a standard curve of L-serine (12.5–100 mg/mL). Then, the extent of *readily bioaccessible protein* formed upon digestion could be expressed as stated in Equation (2).

(2) $$Readily\;bioaccessible\;protein\;\left(\%\right)=\frac{NH_{2TCA}-NH_{2initial}}{NH_{2total}}\times 100$$

### 2.6. Kinetic Modeling and Statistical Analysis 

Mean composition values were calculated based on duplicate measurements. Significant differences between the mean starch, protein, and moisture contents were assessed using one-way ANOVA and Tukey’s HSD tests (*p* < 0.05) (JMP16, SAS Institute Inc., Cary, NC, USA). The starch-to-protein ratio was calculated from the obtained means as an indication of the protein matrix surrounding starch and possibly hindering amylolysis.

Each single digestion tube (13 for the small intestinal phase, cfr. Section 2.4.3) represents an independent evaluation of the same system at a different digestion time [57], as recently reviewed [58]. Applying this multi-tube kinetic approach resulted in the description of the nutrient digestibility of each sample based on 13 independent evaluations. Through regression (see below), all experimental data of one sample type at different kinetic points were integrated. Subsequently, the digestive behavior of different samples was compared in terms of the obtained modeled curves and the estimated parameters. During the small intestinal phase, macronutrient digestion could suitably be fitted with a first-order fractional conversion model (Equation (3)), as previously reported in the context of starch [59] and protein [36] digestion. *C_f_*, the estimated final extent of starch/protein hydrolysis at extended digestion times, and *k*, the estimated reaction rate constant, were simultaneously estimated using kinetic modeling.

(3) $$C\left(t\right)=C_{f}+\left(C_{0}-C_{f}\right)*e^{-k*t}$$

In Equation (3), *C* represents the extent of macronutrient hydrolysis at digestion time *t*. *C_0_* is the hydrolysis extent at the beginning of the small intestinal phase (*t* = 0) (or end of the gastric phase). *C*_f_ and *k* were simultaneously estimated by nonlinear regression (SAS version 9.4, SAS Institute, Inc., Cary, NC, USA). For starch digestion, the model could be simplified (*C*_0_ = 0) with salivary amylase omitted from the in vitro digestion protocol. R^2^_adj_ and residual and parity plots were used to evaluate the fit of the modeled curves. Since consecutive evaluations of one system were used to estimate *k* and *C_f_,* the standard error of those parameters is the result of the uncertainty of the independent measurements. Significant differences between estimated parameters were verified using 95% confidence intervals. 

To take into consideration the correlation between simultaneously estimated parameters *C_f_* and *k*, statistically significant differences among these jointly estimated parameters were evaluated using the Joint Confidence Regions (JCR) analysis (α = 0.05). The slope of the tangent to the modeled curve (for *t* = 0) was determined as the estimated initial rate of macronutrient digestion (v_initial_) [33].

To assess the correlation between amylolytic and proteolytic processes, the digestion data was normalized by dividing each data point by the estimated *C_f_* value, after which normalized digestion curves were modeled and plotted.

The repeatability of the complete experimental set-up used in this work, starting from processing over the enzyme batch characterization and in vitro evaluation of digestion kinetics, was checked and is shown in Supplementary Materials Figure S2 and Table S1.

## 3. Results and Discussion

### 3.1. Characterization of Cooked Lentils and Derived Isolated Cells

#### 3.1.1. Microstructural Characterization

The PSD of isolated cotyledon cells (ICC) and disintegrated cooked lentils (CL), as affected by thermal treatment time followed by mechanical disintegration, are shown in Figure 2. PSDs of ICC fractions were unimodal, indicating that this fraction contained one homogenous fraction of individual cells with sizes between 40 and 400 µm. From this data, assuming spherical particles, the volume-weighed median particle size (d_50_) was 110 µm, 107, and 106 µm for cells isolated from lentils cooked for 15, 30, and 60 min, respectively. It could therefore be concluded that the estimated size of individual cells was independent of the cooking time (confirmed qualitatively by microscopy). These values are in line with previously reported diameters for lentil cotyledon cells of 101 µm [60].

For mechanically disintegrated cooked lentils (CL), a unimodal peak with an average size of around 100 µm could be discerned as well, indicating the presence of individual cotyledon cells. However, next to individual cells, the PSD of cooked lentils was also characterized by both a smaller and a larger fraction. The tail towards smaller sizes (<40 µm) implies the presence of some cellular material released due to breakage (i.e., cell wall fragments, starch granules, and proteins). For example, dimensions of 14–35 µm have been reported for lentil starch granules [60,61]. The fraction of particles larger than individual cells (>400 µm) represents tissue fragments, such as cell clusters and seed coat fragments, as previously reported [18]. Via wet sieving, the fraction (or yield) of individual cotyledon cells was determined to be approximately 48, 50, and 67 g/100 g (dry matter basis) for lentils cooked for 15, 30, and 60 min, respectively. A similar yield of 63% was reported for cell isolation from green lentils for one particular cooking time [19]. Accordingly, the PSDs of mechanically disintegrated CL (Figure 2a) showed an increasing volumetric contribution of individual cells with increasing cooking times and a slight decrease in the other (smaller and larger) fractions. From these PSDs, the fraction of individual cells in the mechanically disintegrated CL cooked for 15, 30, and 60 min could be calculated to be 43.5 ± 5.6, 48.6 ± 3.7, and 49.2 ± 1.3%, respectively. The volumetric contributions of the cellular (40–400 µm), subcellular (broken cell material, <40 µm), and super cellular (larger cell cluster and tissue fraction, >400 µm) to the total volume of the CL samples are shown in Figure 2b. While the differences in the cellular fraction calculated based on PSDs are not statistically significant (*p* < 0.05) for different cooking times, an increasing trend of the cellular fraction with the increasing cooking times was observed for the cellular yield obtained based on wet sieving. These results indicate that shorter cooking times lead to more cell breakage upon mechanical disintegration, on the one hand, and to the presence of more clusters of cotyledon cells (intact tissue fragments) on the other hand. In contrast, longer cooking times lead to more complete cell separation and thus a larger (volumetric and mass) fraction of individual cells.

#### 3.1.2. Compositional Characterization

The macronutrient compositional analysis (Table 1) revealed no significant differences in the protein contents between raw and whole CL (~22 g/100 g DM). In the literature, similar protein (21 to 29 g/100 DM) and starch (46 to 50 g/100 g DM) contents were reported for cooked whole lentils [43,62]. Shifts in a composition can be explained by the net leaching of dry matter during soaking and cooking, followed by discarding of those liquids. For ICC, a protein content of 20–21 g/100 g DM was measured, significantly lower as compared to uncooked lentils but not significantly affected by the applied cooking time. The starch content of CL was slightly lower as compared to raw lentils, showing an increasing trend with the cooking time (between 45 and 47 g/100 g DM), but these differences were not statistically significant. The ICC showed a significantly higher starch content (61 to 62 g/100 g DM) as compared to raw and cooked whole lentils, with no significant effect of the cooking time.

Differences in the overall composition between ICC and CL samples can be explained by the process of wet sieving, leading to the concentration of cotyledon cells (which are rich in starch), while other fractions are discarded (especially fiber-rich hulls). Literature reports on lentil ICC are missing, but similar protein and starch contents were reported for the ICC of other pulses (e.g., peas and common beans) [31,33]. Generally, in terms of (nutrient) composition, the consumption of whole CL (or food ingredients derived thereof) rather than ICC (or powdered fractions enriched in intact cotyledon cells) could be interesting in view of increasing the protein and fiber intake. 

Overall, a starch-to-protein ratio of 2.2 was calculated for raw lentils. For CL and ICC, starch-to-protein ratios between 2.0 and 2.1 and 3.0 and 3.1 were found, respectively. A lower starch-to-protein ratio indicates that more protein surrounds starch, which could potentially exert a barrier effect hindering amylolysis. In comparison, similar starch-to-protein ratios were reported for whole cooked chickpea and corresponding ICC, while lower ratios were reported for black beans (seeds and ICC) [31].

The calorimetric analysis (Supplementary Materials Figure S1) showed complete starch gelatinization in all samples (cooked lentils and isolated cells with different cooking times). Therefore, possible differences in starch digestibility between samples can be attributed to the abovementioned process-induced differences (i.e., microstructure and composition) rather than differences in the starch gelatinization degree and preserved native organization.

### 3.2. Effect of Cooking Time on In Vitro Amylolysis

**Figure 3:**
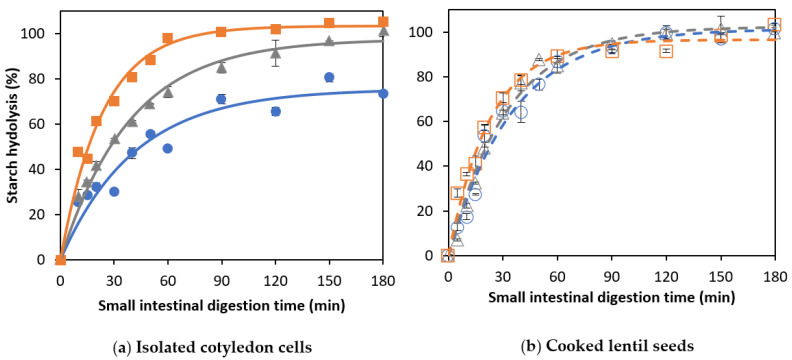
In vitro amylolysis as a function of the small intestinal digestion time of (**a**) cells isolated from lentils cooked for (○)15, (∆) 30, or (□) 60 min and (**b**) whole cooked lentils cooked for (●) 15, (▲) 30, or (■) 60 minutes. Symbols represent experimentally determined data. Lines represent the data modeled using the fractional conversion model (Equation (3)). Error bars indicate the standard deviation of the analytical replicates.

Amylolysis during the in vitro digestion of CL and corresponding ICC is shown in Figure 3. 

#### 3.2.1. Amylolysis Kinetics of Isolated Lentil Cotyledon Cells (ICC)

For ICC, starch hydrolysis followed a similar trend for all applied cooking times, reaching complete starch hydrolysis at the end of the simulation (Figure 3a). The experimental data were fitted using a fractional conversion model, with the estimated model parameters shown in Table 2. 

**Figure 4:**
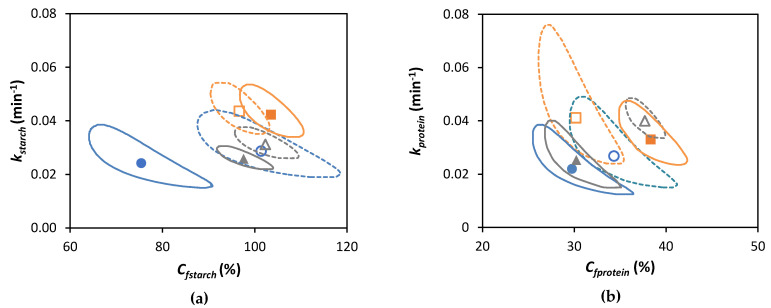
Joint Confidence Region (JCR) analysis (95% confidence level) of jointly estimated kinetic parameters estimated using the fractional conversion model (Equation (3)) for (**a**) in vitro amylolysis and (**b**) in vitro proteolysis of cooked lentils (●, ▲, and ■) and corresponding isolated cotyledon cells (○, ∆, and □) cooked for 15, 30, or 60 min, respectively. Full and dotted lines represent JCR of whole cooked lentils and isolated cotyledon cells, respectively.

For lentil ICC, no significant effect of the cooking time on the final extent (*C_f_*) of starch digestion could be observed (confirmed by JCR analysis, Figure 4a). Similar observations were made for Bambara groundnuts [32], chickpeas, and green peas [31]. In contrast, an increase in the final starch digestibility was found with increasing cooking times for cells isolated from common beans and attributed to changes in cell wall permeability [21]. Although amylolysis followed similar general trends for lentil ICC with different cooking times, Table 2 shows that both the rate constant (*k*) and initial rate of starch hydrolysis (v_initial_) increased as a function of the cooking time (linear correlations with R^2^ of 0.97 and 0.99, respectively, data shown in Supplementary Materials Figure S3). 

An increase of v_initial_ with increasing cooking times indicates a possible decrease of the physical barrier exerted by the cell (wall and protein matrix), relatively facilitating amylases reaching their substrate. An increase of *k* with the cooking time implies that the reaction plateau (*C_f_*) will be reached faster during digestion. Similar trends have been observed for ICC of several other pulse types [31,32,33]. Regardless of cooking time, amylolysis in lentil ICC followed a fractional conversion model, with the rate of amylolysis decreasing with the proceeding digestion time. In contrast, for some other pulse ICC (e.g., common beans, black beans, and Bambara groundnuts), a lag phase, characteristic for the time needed for enzymes to reach the substrate, could be discerned [31,32,33]. These differences in the amylolysis kinetics between lentil ICC and, for example, black and common bean ICC, could possibly be explained by differences in multiple cellular properties [34]. For example, black bean ICCs have a lower starch-to-protein ratio (2.3–2.4) [31] and thus possibly a thicker protein matrix surrounding starch compared to lentils (3.0–3.1). Additionally, differences in amylolysis kinetics could be attributed to cell wall differences as well, with lentils possessing a lower pectin content (19.4–22.3%) compared to, e.g., common beans (28.5–41.2%) [63,64]. In this context, in common beans, gradual pectin solubilization (as a function of the cooking time) has been linked to the gradually increasing permeability of the cell wall to digestive amylases and, consequently, a shorter lag phase [33]. Since no lag phase was observed for lentil ICC, similar to previously reported results on Bambara groundnuts ICC [32], it could be hypothesized that pectin solubilization might be less cooking time-dependent compared to common beans. Possibly, in lentil ICC, permeation through the protein matrix (entrapped within the cell wall), rather than the permeability and permeation through the cell wall itself, might be a major factor affecting the amylolysis rate (v_initial_ and *k*). In this case, gradual protein hydrolysis would be expected to play an important role affecting amylolysis kinetics, and factors facilitating proteolysis could also facilitate amylolysis, as discussed in Section 3.4. From this discussion, it can generally be concluded that ICC amylolysis kinetics and how these are affected by the cooking time differ distinctly between pulse types. The underlying reasons could be elucidated by studying different pulse cell properties (e.g., composition and cell wall permeability) and correlating them to established digestion kinetics.

#### 3.2.2. Amylolysis Kinetics of Whole Cooked Lentil Seeds (CL)

As for ICC, amylolysis in CL followed a fractional conversion behavior (Figure 3b), with the estimated parameters shown in Table 2. Mechanically disintegrated CL clearly showed an increasing trend of *k* and *C_f_* with an increasing cooking time. Moreover, as can be seen from Table 2, the cooking time was clearly impacted v_initial_ (linear relation with R^2^ of 0.995, data shown in Supplementary Materials Figure S3), as well as *C_f_* and *C*_120_. 

CL with a shorter cooking time (15 min) showed a significantly (*p* < 0.05) lower plateau value (75.4 ± 5.4%) compared to ICC with the same cooking time (101.4 ± 3.2%). Differences in amylolysis kinetics between cooked lentils with different cooking times can be attributed to differences in PSD upon mechanical disintegration (Figure 2). Mechanically disintegrated lentils cooked for 15 min contained slightly more cell clusters (52%) notably delaying amylolysis compared to lentils cooked for 30 and 60 min (48 and 47%, respectively). However, upon longer cooking times (30 and 60 min), the final starch digestion extents for CL were not significantly different from their corresponding ICC, all reaching approximately complete amylolysis upon long simulated digestion times (confirmed by JCR analysis, Figure 4a). Cell separation upon mechanical disintegration was more complete with these longer cooking times, resulting in more individual cells (48 and 49% based on the volumetric PSD and 50 and 67% on based on mass) compared to CL with a cooking time of 15 min (43% and 48%, respectively). In other words, lentils with a short cooking time showed a significantly attenuated amylolysis compared to the corresponding ICC. Upon increasing the cooking time, however, starch digestion in whole lentils approached the behavior of their most characteristic fraction, namely individual cotyledon cells. Similar overall trends were previously reported for other pulse types such as common beans [21] and Bambara groundnuts [32].

Another factor possibly contributing to the low starch digestibility of lentils cooked for a short time (15 min) compared to the isolated cells of the same cooking time could be the presence of the seed coat, which is rich in dietary fiber and polyphenols, which can interfere with starch digestion [23,65,66]. Upon longer cooking times, the interactions between polyphenols and starch could be broken, rendering the effect on starch digestibility negligible [67]. In that regard, the total amount of polyphenols in lentils has been shown to reduce 56% upon cooking times longer than 30 min [68]. Moreover, in the literature on other pulse types, partial (gastric) degradation of the intracellular protein matrix entrapping starch has been related to facilitated amylolysis [54]. Possibly, increasing denaturation of the protein matrix surrounding starch with increasing cooking time could facilitate (gastric) proteolysis [69,70,71]. In turn, a more degraded intracellular protein matrix could facilitate small intestinal amylolysis. 

The insights gathered in this work could be used to create foods (and food ingredients) with macronutrient digestibility, depending on consumer requirements. For example, while bioencapsulation generally attenuates amylolysis in cooked lentil seeds, consuming lentils with a shorter cooking time (15 min) could be preferred if an even slower release of starch digestion products is targeted (e.g., low glycemic index).

It should be noted that measured values for starch hydrolysis, as well as the modeled final value *C_f_*, slightly exceeded 100% in some cases. This is not realistic but could be explained by the applied spectrophotometric DNS assay (in terms of maltose equivalents). During starch hydrolysis by amylases, mostly maltose and maltotriose are formed [72,73]. Other hydrolysis products, such as glucose, can be formed as well [74], causing an overestimation of the extent of hydrolysis [73]. Alternative methods to specifically quantify different starch hydrolysis products (rather than only maltose) have been implemented, such as glucose quantification (e.g., GOPOD) [35,60,73] and HPAEC-PAD [36]. Generally, differences in terms of the total starch digestibility were observed, depending on the applied method [36,73], reaching around 10% in the case of DNS compared to HPAEC-PAD. However, the delivered overall trends (and rate constants) were similar. In this context, it should be noted that in vitro digestion assays are not suited to predict the exact extent of enzymatic macronutrient digestion in vivo [58,73]. Rather, in vitro digestion can deliver insights into digestive patterns and rates at a given (fixed) enzyme activity and allow the comparison and evaluation of digestion patterns of different samples under these fixed conditions [73]. After all, a lower rate of amylolysis in vitro could be hypothesized to lead to a (s)lower release of glucose into the bloodstream and a higher probability of (more) undigested starch reaching the colon [58,73]. Therefore, despite being a “black box” approach in terms of the formed metabolites, the DNS method remains a widely applied, rapid, and easy-to-use and suitable method for comparing (pulse) samples with different processing histories in terms of the amylolysis pattern [31,32,75,76].

### 3.3. Effect of Cooking Time on In Vitro Proteolysis 

**Figure 5:**
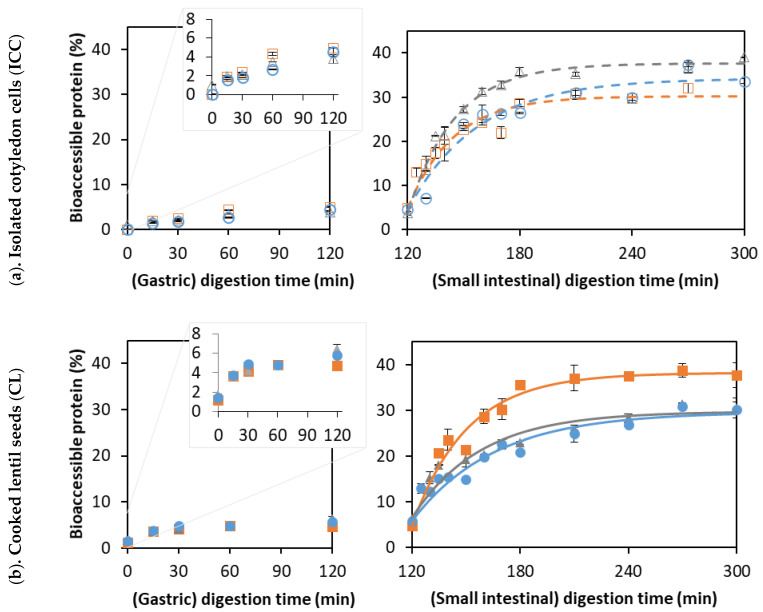
The formation of readily bioaccessible proteins as a function of the digestion times of (**a**) isolated lentil cells derived from lentils cooked for (○) 15, (∆) 30, and (□) 60 min, and (**b**) whole lentil seeds cooked for (●) 15, (▲) 30, and (■) 60 min. Symbols represent experimentally determined data. Lines represent the data modeled using the fractional conversion model (Equation (3)). Error bars indicate the standard deviation of analytical replicates.

The formation of readily bioaccessible protein (NH_2TCA_) upon the in vitro digestion of CL and corresponding ICC is shown in Figure 5. 

For all samples, only about 5% readily bioaccessible proteins were formed during the gastric phase, which is in accordance with the literature on other pulses such as common beans and soybeans [53,54]. This can be explained by the specificity of pepsin, preferably cleaving peptide bonds between aromatic amino acids such as phenylalanine, tryptophan, and tyrosine [77]. Pulse proteins are typically poor in sulfur-containing amino acids and tryptophan [78], explaining their lower susceptibility to pepsinolysis. In contrast, lentil proteins seem to be a much more ideal substrate for small intestinal trypsin and chymotrypsin, which preferably cleave at C-terminal basic amino acids (e.g., arginine and lysine) and/or large hydrophobic residues (e.g., phenylalanine and tyrosine) [77,79]. 

The experimental data of the small intestinal phase were modeled using a fractional conversion model with the estimated parameters shown in Table 3 and JCR analysis in Figure 4b.

#### 3.3.1. Proteolysis Kinetics of Isolated Lentil Cotyledon Cells (ICC)

For ICC, the final levels of readily bioaccessible proteins of 30 to 37% were estimated. In comparison, final values of 35–36% were reported for the in vitro digestion of black bean and chickpea ICCs [31], and values of around 50% were found for common bean ICC [54]. However, the initial reaction rate (v_initial_) was significantly higher for isolated lentil cells with a cooking time of 30 min compared to both the shorter (15 min) and longer (60 min) cooking times. An increase in the initial hydrolysis rate with the cooking time could be explained by increased protein denaturation increasing the susceptibility to proteases [69,70] and a decrease in the antinutrient content (e.g., trypsin inhibitors) with increasing cooking times [80,81]. Similarly, an increase in vitro protein digestibility has been reported upon cooking for several pulses [71]. 

However, the *C_f_* was significantly lower for isolated cells with a cooking time of 60 min compared to a cooking time of 30 min, confirming that protein digestibility may decrease upon prolonged cooking. Further increasing the cooking time (to 60 min) could possibly decrease the susceptibility of protein-to-enzymatic hydrolysis, as seen previously for Canavalia [82]. The decrease in digestibility upon long cooking times could potentially be attributed to protein aggregation through the formation of intra- and intermolecular interactions [71,83]. In contrast, other research on protein digestibility in Bambara groundnuts did not show this trend as a function of the cooking time. Therefore, interactions occurring between proteins upon (thermal) processing and the consequences for digestibility should be studied further, for example, using molecular techniques such as SEC-HPLC [84].

#### 3.3.2. Proteolysis Kinetics of Whole Cooked Lentil Seeds (CL)

For whole cooked lentils, an increasing trend could be observed for the *k*, *C_f_*, and v_initial_ values with increasing the cooking time (Table 3), indicating a facilitation of proteolysis upon thermal treatment. Next to the abovementioned effects (protein denaturation and inactivation of antinutrients), the PSD upon mechanical disintegration could also play a role here (Figure 2). Upon shorter cooking (i.e., 15 min), cell separation was less complete, and more cell clusters remained present, which could, in turn, result in slower and less complete protein digestion. In this context, delayed proteolysis was observed for large navy bean flour particles (made up of cell clusters) and clusters of soybean cells [55,85]. 

For a short cooking time (15 min), all estimated modeling parameters were significantly lower for CL compared to the corresponding ICC. In contrast, for a cooking time of 60 min, no significant difference in *k* and v_initial_ could be discerned between the CL and ICC. As mentioned for the case of starch digestion (Section 3.2), the PSD of whole lentils with a cooking time of 60 min closely approached that of ICC. These findings support the importance of the process-induced microstructure in determining protein digestibility. Additionally, polyphenols (present in the seed coat of whole lentils) can slow protein digestion by forming complexes with proteins [86] but can leach out during cooking, which could lower their concentration upon longer cooking times [68].

It should be noted that, when using the OPA analysis, only the terminal amino groups of free amino acids and di- and tripeptides were quantified. In vivo, these small peptides can be readily absorbed through the intestinal epithelial wall. The applied method does not make a distinction between amino acids and di- and tripeptides, and the obtained results are difficult to relate directly to the degree of hydrolysis. More information could therefore be obtained by hydrolyzing the TCA-soluble (small peptide) fraction, quantifying the bioaccessible proteins in terms of the amino acid constituents [18,29]. Moreover, larger soluble protein hydrolysates were not quantified by this method, though these could be hydrolyzed by brush border enzymes in vivo. Quantification of these alternative fractions led to different (i.e., higher) proteolysis extents, but overall, similar trends and differences between samples could be discriminated [18,29,32]. Taking this into account, the quantification of TCA-soluble bioaccessible proteins (NH_2TCA_) remains a useful, sensitive, and high-throughput method of analysis [87]. It is a much-applied parameter for comparing the protein digestibility of differently processed samples in general terms [29,32,54,55,88].

### 3.4. Qualitative Evaluation of Amylolysis and Proteolysis Using Microscopic Observations

**Figure 6:**
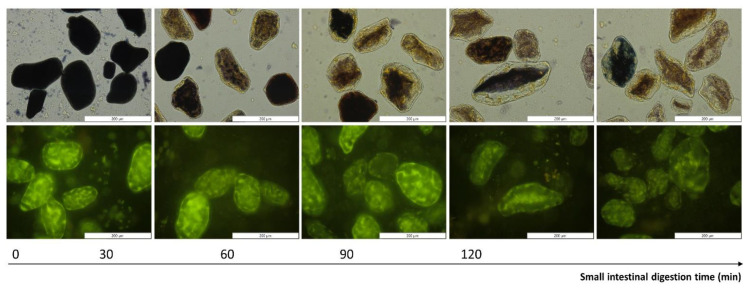
Representative micrographs of digested pellets of isolated cotyledon cells (with a cooking time of 30 min) as a function of small intestinal digestion time. First row: gradual amylolysis was visualized by staining with Lugol (dark blue). Second row: proteolysis was visualized using (green) fluorescence microscopy (exposure time 500 ms). A magnification of 40× was utilized and the scale bars represent a length of 200 µm.

Changes occurring upon in vitro digestion were shown qualitatively using representative micrographs of pellets obtained upon centrifugation of the digests (cfr. Section 2.4.3) and are shown for one sample (ICC with a cooking time of 30 min) in Figure 6. Starch digestion was visualized upon staining of the lentil pellets with Lugol’s iodine reagent. Polyiodide ions interact with amylose, forming a dark blue-to-black color. The higher the starch polymerization degree, the higher this interaction and the more intense the formed color [89]. At the start of the small intestinal phase, no amylolysis had occurred, and the cells in the digestion pellets appeared dark. With increasing the digestion time, enzymatic amylolysis caused a decrease in the starch polymerization degree, gradually causing a shift in the observed color to lighter blue and even brown-yellowish colors. From these micrographs, it could clearly be observed that cells remained intact upon small intestinal digestion and were gradually radially emptied. This confirmed that amylolysis occurred inside cotyledon cells upon the permeation of pancreatic amylase through the cell wall and intracellular protein matrix, as reported for other pulse ICCs [24,27,33,60]. Interestingly, upon long digestion times (*t* = 120 min), some cells still showed a dark color, indicating these were little affected by pancreatic amylase, as reported previously for, e.g., Bambara groundnuts [32].

Protein disappearance could be evaluated qualitatively as well by observing the decrease in intrinsic fluorescence of aromatic amino acids present in the digestion pellets upon digestion [46]. In these images, starch could be observed as dark spots surrounded by the fluorescent protein matrix [47,54]. Similar protein disappearance patterns were previously reported for other pulse types [5,7,32].

### 3.5. Linking Starch and Protein Digestion Kinetics 

The (cytoplasmatic) protein matrix present inside pulse cotyledon cells has been demonstrated to carry out a barrier effect hindering starch digestion but not (yet) for the case of lentils [31,32,54,60]. Research on common beans and chickpeas revealed that gastric (pepsin) proteolysis had a significant role facilitating small intestinal amylolysis in common beans [29,54]. 

**Figure 7:**
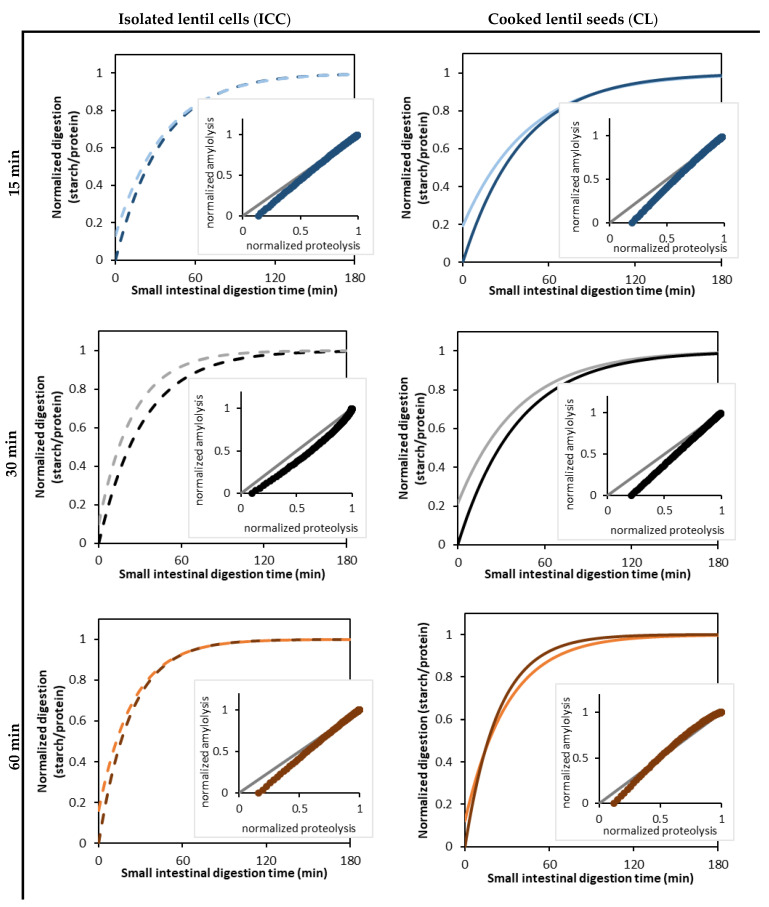
Normalized parameter estimates of readily bioaccessible protein (bright color) formation and amylolysis (dark color) kinetics during the in vitro small intestinal digestion of isolated cotyledon cells (ICC, left, dotted lines) and whole cooked lentils (CL, right, full lines) cooked for 15 (**-**/**-**), 30 (**-**/**-**), and 60 (**-**/**-**) minutes. The kinetic parameters for amylolysis and proteolysis were estimated by fitting the experimental data to a fractional conversion model (Equation (3)). The inserted scatter shows the correlation analysis of amylolysis and proteolysis, with the grey diagonal (**-**) indicating the case of identically progressing proteolysis and amylolysis.

The correlation analysis revealed a positive correlation between the estimated rate constant of amylolysis and proteolysis, as well as the final extent of starch and protein digestion (data in Supplementary Materials Figure S4). For example, for the CL, the rate constant of amylolysis and proteolysis showed a strong linear correlation (R^2^ of 0.95). To further assess the correlation between these two hydrolytic processes, the starch and protein digestion kinetic data were normalized (Figure 7). This data showed that proteolysis slightly preceded amylolysis due to pepsinolysis during the gastric phase, during which starch remained unaffected. A strong (almost linear) relation was observed between starch and protein digestion in the small intestinal phase for both the CL and ICC and for all applied cooking times. It could thus be observed that amylolysis and proteolysis occurred simultaneously, while amylolysis seemed to occur slightly faster than proteolysis. Similar relations between starch and protein hydrolysis upon in vitro digestion have been reported earlier for chickpeas [31]. Based on the literature stated above, this indeed indicates that the proceeding protein hydrolysis could facilitate amylolysis.

For the ICC, no effect of the cooking time could be discerned on the increase of amylolysis with proceeding proteolysis. This indicates that the way proteolysis affects amylolysis in cotyledon cells is not likely dependent on the applied cooking time. Indeed, similar amylolysis kinetics were observed for the ICC upon different cooking times (Section 3.2), and small but significant differences in proteolysis kinetics could be discerned (Section 3.3).

In the future, the trends identified in this work for lentils and elsewhere for other pulse types can be compared and correlated to their microstructural and compositional properties to unravel the properties and mechanisms governing pulse digestion kinetics. 

## 4. Conclusions

In this study, the effect of the cooking time within the palatable range (15, 30, and 60 min) on microstructural properties (tissue failure mode) and related to the in vitro digestion functionality of cooked lentil seeds (CL) and cotyledon cells isolated thereof (ICC) were evaluated. Regardless of cooking time, isolated cotyledon cells showed similar amylolysis kinetics and small but significant (*p* < 0.05) differences in proteolysis kinetics. In contrast, larger differences in the digestion kinetics of whole lentils could be obtained as a function of the applied cooking time. In this work, the presence of higher fractions of cell clusters upon mechanical disintegration were linked to (s)lower starch and protein digestion kinetics for whole lentils with a shorter cooking time (i.e., 15 min) compared to longer cooking times. The obtained digestion kinetics and correlation analysis indicated that amylolysis and proteolysis occurred simultaneously, while amylolysis seemed to occur slightly faster than proteolysis. Although the proceeding proteolysis might facilitate amylolysis, the rate of amylolysis in CL and ICC seems to be mostly determined by differences in the microstructural organization as a result of the applied cooking time. 

Future research can be directed towards the comparison and correlation of (microstructural and compositional) pulse properties and their digestive behavior to better understand the mechanisms and factors determining macronutrient digestion in pulses. Moreover, this work focused solely on macronutrient release in the upper gastrointestinal tract, revealing the presence of (encapsulated) starch and protein in the non-bioaccessible pellet fraction at the end of small intestinal digestion. Research into the subsequent colonic fermentation of lentils is an important next step.

The results of this work highlight that the digestive functionality of lentils depends on the (intensity of the) applied processing steps. Depending on the targeted digestive functionality, the applied cooking time can be adapted to either optimize the energy (starch) utilization from the seeds (longer cooking times) or, in contrast, minimize the glycemic index (shorter cooking times). As well as slowed amylolysis, based on the research on other pulse types, the application of a short cooking time within the palatable range could lead to improved mineral bioaccessibility as well [90,91], but research in this area is currently missing. 

The current insights can also be used to optimally exploit the nutritional potential of lentils, for example, by formulating population-specific recommendations for lentil preparation (at the household level). Additionally, this work highlighted the potential of innovative (lentil) ingredients with (partial) cellular intactness for the development of foods with steered digestive functionality, preferably using the whole pulse seeds rather than isolated cellular fractions. 

## Figures and Tables

**Table 1:** Macronutrient composition (in g/100 g dry matter) of whole cooked lentil seeds and cotyledon cells isolated thereof as a function of the applied cooking time. Reported values are means of duplicate measurements ± standard deviation. Within a column, different superscript letters indicate significant differences between the reported means (*p* < 0.05).

	Moisture (g/100 g)	Starch (g/100 g DM)	Protein (g/100 g DM)	Starch-to-Protein Ratio
Raw lentil	9.4 ± 0.4 ^d^	48.3 ± 1.0 ^b^	22.3 ± 0.4 ^a,b^	2.17
**Isolated cotyledon cells** (**ICC**)				
15 min	73.0 ± 0.1 ^b^	62.2 ± 1.0 ^a^	20.6 ± 0.1 ^c,d^	3.02
30 min	73.1 ± 0.2 ^b^	60.8 ± 1.5 ^a^	19.8 ± 0.7 ^d^	3.06
60 min	76.1 ± 0.1 ^a^	62.6 ± 1.7 ^a^	21.2 ± 0.4 ^b,c,d^	2.96
**Cooked lentil seeds** (**CL**)				
15 min	68.4 ± 0.7 ^c^	44.7 ± 2.2 ^c^	21.7 ± 0.1 ^a,b,c^	2.06
30 min	70.1 ± 0.1 ^c^	45.9 ± 1.6 ^b,c^	22.7 ± 0.1 ^a^	2.02
60 min	75.9 ± 1.0 ^a^	46.9 ± 0.8 ^b,c^	22.3 ± 0.2 ^a,b^	2.10

DM: Dry matter.

**Table 2:** Kinetic parameters (± standard deviation) estimated using the fractional conversion model (Equation (3)) for the in vitro amylolysis of whole cooked lentil seeds (CL) and isolated cotyledon cells (ICC) for different cooking times. After the rate constant *k* and final extent of amylolysis *C_f_*, the initial amylolysis rate (v_initial_) and amylolysis extent at 120 min of the small intestinal simulation (C_120_) were estimated. Within a column, different letters in superscript indicate significant difference between means based on 95% confidence intervals.

Cooking Time	v_initial_ (%/min)	*k* (/min)	*C_f_* (%)	C_120_ (%)	R^2^_adj_
**Isolated cotyledon cells** (**ICC**)					
15 min	2.9 ± 0.3 ^b^	0.029 ± 0.003 ^c^	101.4 ± 3.2 ^a^	98.2 ± 2.4 ^a,b^	0.99
30 min	3.2 ± 0.2 ^b^	0.031 ± 0.002 ^b,c^	102.3 ± 2.5 ^a^	99.8 ± 2.0 ^a,b^	0.99
60 min	4.2 ± 0.3 ^a^	0.044 ± 0.003 ^a^	92.8 ± 2.1 ^a^	92.3 ± 2.0 ^b^	0.99
**Cooked lentil seeds** (**CL**)					
15 min	1.8 ± 0.3 ^d^	0.024 ± 0.003 ^c^	75.4 ± 4.3 ^b^	71.3 ± 2.9 ^c^	0.99
30 min	2.5 ± 0.2 ^c^	0.026 ± 0.001 ^c^	97.6 ± 2.1 ^a^	93.2 ± 1.4 ^b^	0.99
60 min	4.3 ± 0.4 ^a^	0.042 ± 0.003 ^a,b^	103.5 ± 2.39 ^a^	102.8 ± 2.2 ^a^	0.99

**Table 3:** Kinetic parameters (± standard deviation) estimated using the fractional conversion model (Equation (3)) for in vitro proteolysis of whole cooked lentil seeds (CL) and isolated cotyledon cells (ICC) for different cooking times. The estimated kinetic parameters include the rate constant *k*, final extent of proteolysis *C_f_*, and the initial proteolysis rate (v_initial_). Within a column, different letters in superscript indicate significant differences between means based on 95% confidence intervals.

Cooking Time	v_initial_ (%/min)	*k* (/min)	*C_f_* (%)	R^2^_adj_
**Isolated cotyledon cells** (**ICC**)				
15 min	0.798 ± 0.177 ^c^	0.027 ± 0.005 ^a^	34.32 ± 1.82 ^a,b^	0.99
30 min	1.356 ± 0.186 ^a^	0.040 ± 0.003 ^a^	37.68 ± 0.74 ^a^	0.99
60 min	1.083 ± 0.162 ^b^	0.041 ± 0.006 ^a^	30.15 ± 1.25 ^b^	0.99
**Cooked lentil seeds** (**CL**)				
15 min	0.528 + 0.101 ^d^	0.022 ± 0.004 ^a^	29.74 ± 1.67 ^b^	0.99
30 min	0.752 + 0.144 ^c^	0.025 ± 0.004 ^a^	30.21 ± 1.36 ^b^	0.99
60 min	1.109 + 0.137 ^b^	0.033 ± 0.004 ^a^	38.31 ± 1.24 ^a^	0.99

## Data Availability

The data presented in this study are openly available on Zenodo.org at 10.5281/zenodo.7561181.

## Supplementary Materials

The following supporting information can be downloaded at: https://www.mdpi.com/article/10.3390/foods12030525/s1: Figure S1: DSC thermograms obtained for (**A**) lentils cooked for 15 or 60 min, and (**B**) lentil cells isolated from lentils cooked for 15, 30, or 60 min. These thermograms indicate the complete gelatinization of starch due to the absence of a peak around 68–69 °C, with the peak around 55 °C representing the gelatinization of retrograded starch. The peak around 83 °C is probably due to the formation of amylose–lipid complexes and/or residual protein denaturation. Figure S2: Reproducibility of the experimental set-up used in this work evaluated for in vitro amylolysis as a function of the small intestinal digestion time of whole cooked lentils cooked for 30 min (▲), as used in this work (see Figure 3a). (●) and (■) represent evaluations of lentils of the same batch independently cooked, mechanically disintegrated, and in vitro digested in duplicate using separately characterized enzyme batches. Symbols represent experimental data. Lines represent the data modeled using the fractional conversion model (Equation (3)). For the three independent evaluations, the estimated model parameters are not significantly different (*p* < 0.05). Error bars indicate the standard deviation of the analytical replicates. Table S1: Estimated kinetic parameters (±standard error) of the fractional conversion model (Equation (3)) for the in vitro amylolysis of whole lentil seeds (CL) cooked for 30 min. CL30_rep1_ and CL30_rep2_ indicate independent evaluations of lentils of the same batch independently cooked, mechanically disintegrated, and in vitro digested in duplicate using separately characterized enzyme batches. Estimated parameters are the rate constant *k* and final extent of amylolysis *Cf*. Within a column, different letters in superscript indicate significant differences between means based on 95% confidence intervals. The kinetic parameters do not differ significantly over independent repetitions of the digestion experiments, proving the reproducibility of the experimental set-up and digestion evaluations. Figure S3: Estimated model parameters for the amylolysis of (●) whole cooked lentils (CL) and (●) isolated cotyledon cells (ICC) during small intestinal digestion as a function of the applied cooking time: (a) reaction rate constant *k* (/min), (b) initial reaction rate *vi* (%/min), and (c) final extent of hydrolysis *Cf* (%). Error bars indicate 95% confidence intervals of the estimated parameters. Figure S4: Correlation between the estimated model parameters for amylolysis and proteolysis of (●) whole cooked lentil seeds (CL) and (●) isolated cotyledon cells (ICC) during small intestinal digestion: (a) reaction rate constant *k* (/min) and (b) final extent of hydrolysis *Cf* (%). Error bars indicate 95% confidence intervals of the estimated parameters.

## Abbreviations

ICC—Isolated cotyledon cells; CL—Whole cooked lentil seeds; PSD—Particle size distribution; DSC—Differential Scanning Calorimetry; SSF—Simulated Salivary Fluid; SGF—Simulated gastric fluid; SIF—Simulated intestinal fluid.
