# Supplementary material for: How Cooking Time Affects In Vitro Starch and Protein Digestibility of Whole Cooked Lentil Seeds versus Isolated Cotyledon Cells
Source: Foods. 2023 Jan 24;12(3):525. doi: 10.3390/foods12030525 (PMC9914867; doi:10.3390/foods12030525)
Supplement: Supplementary file 1 [file foods-12-00525-s001.zip › foods-2132207-supplementary.pdf]

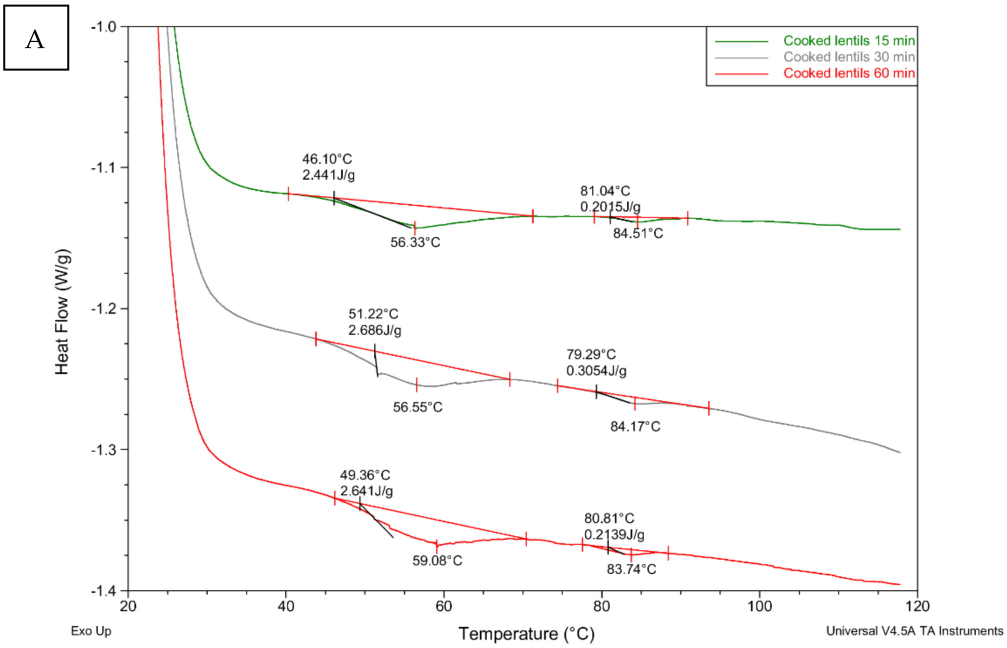

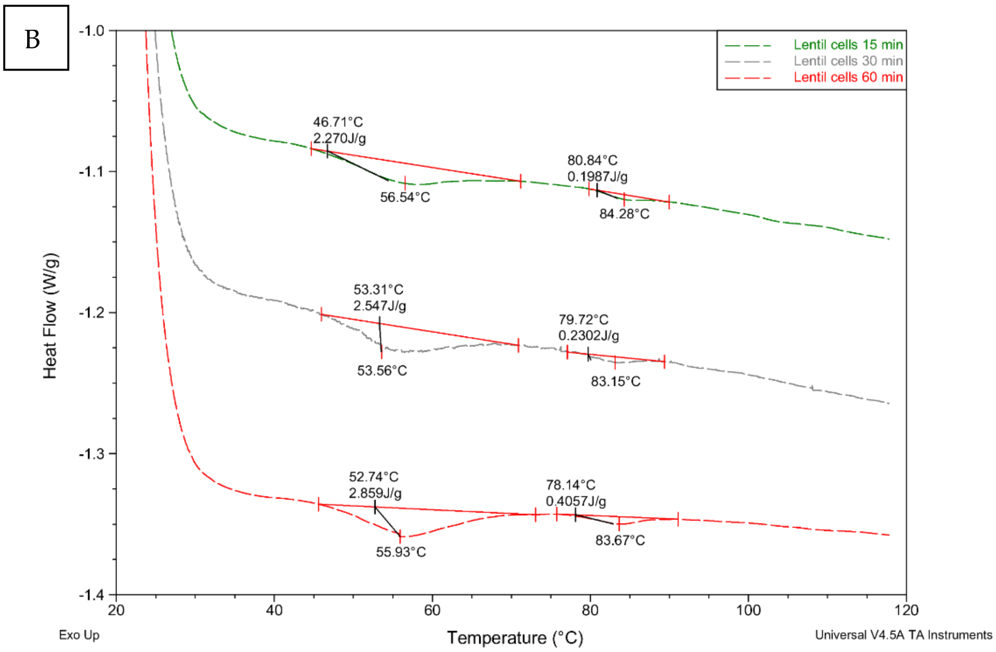

Figure S1. DSC thermograms obtained for (A) lentils cooked for 15, or 60 minutes, and (B) lentil cells isolated from lentils cooked for 15, 30, or 60 minutes. These thermograms indicate complete gelatinization of starch due to the absence of a peak around 68-69 °C, with the peak around 55 °C representing gelatinization of retrograded starch. The peak around 83 °C is probably due to the formation of amylose-lipid complexes and/or residual protein denaturation.

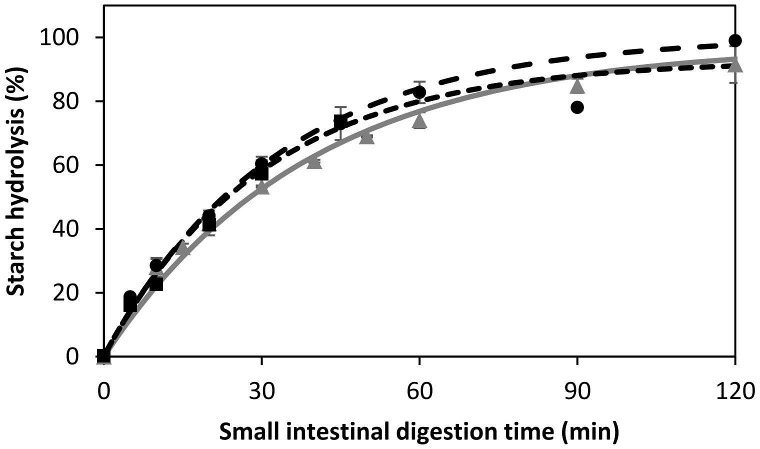

Figure S2. Reproducibility of the experimental set-up used in this work evaluated for in vitro amylolysis as a function of small intestinal digestion time of whole cooked lentils cooked for 30 minutes (▲) as used in this work (see Figure 3 (a)). (●) and (■) represent evaluations of lentils of the same batch, independently cooked, mechanically disintegrated, and in vitro digested in duplicate using separately characterized enzyme batches. Symbols represent experimental data. Lines represent the data modelled using the fractional conversion model (Eq. 3). For the three independent evaluations, the estimated model parameters are not significantly different ($p<0.05$). Error bars indicate the standard deviation of analytical replicates.

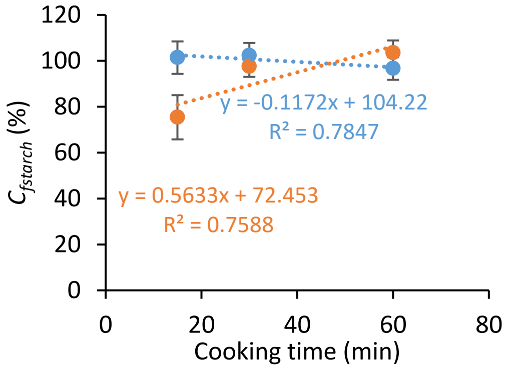

(a)

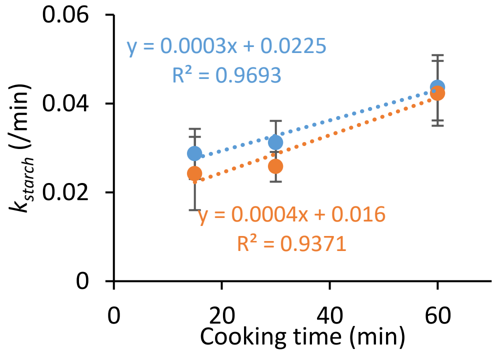

(b)

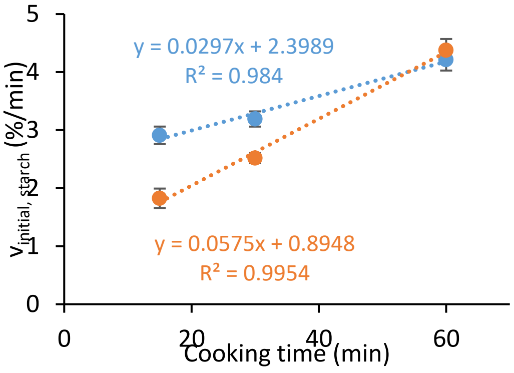

(c)

Figure S3. Estimated model parameters for in vitro amylolysis of (●) whole cooked lentils (CL) and (●) isolated cotyledon cells (ICC) during small intestinal digestion as a function of applied cooking time: **(a)** reaction rate constant *k* (/min), **(b)** initial reaction rate v_i (%/min), and **(c)** final extent of hydrolysis C_f (%).Error bars indicate 95% confidence intervals of the estimated parameters.

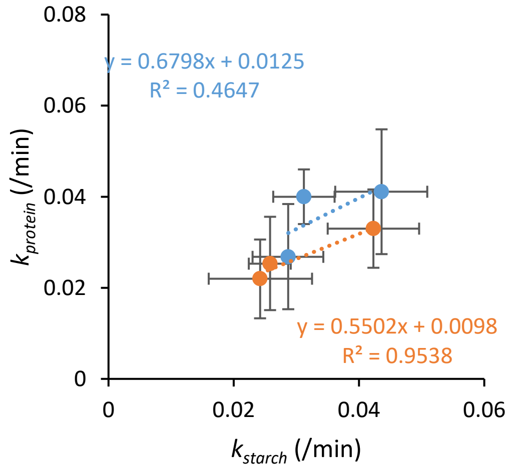

(a)

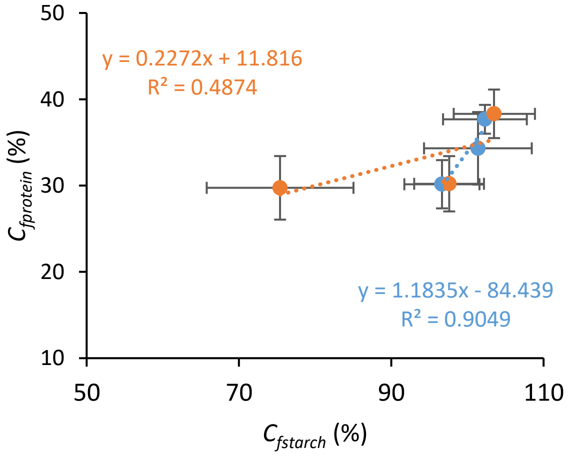

(b)

Figure S4. Correlation between estimated model parameters for in vitro amylolysis and proteolysis of (●) whole cooked lentil seeds (CL) and (●) isolated cotyledon cells (ICC) during small intestinal digestion: **(a)** reaction rate constant *k* (/min), and **(b)** final extent of hydrolysis C_f (%). Error bars indicate 95% confidence intervals of the estimated parameters.

Table S1. Estimated kinetic parameters (± standard deviation) of the fractional conversion model (Eq. 3) for in vitro amylolysis of whole lentil seeds (CL) cooked for 30 minutes. $CL30_{rep1}$ and $CL30_{rep2}$ indicate independent evaluations of lentils of the same batch, independently cooked, mechanically disintegrated, and in vitro digested in duplicate using separately characterized enzyme batches. Estimated parameters are the rate constant *k* and final extent of amylolysis C_f. Within a column, different letters in superscript indicate significant difference between means, based on 95% confidence intervals. The kinetic parameters do not differ significantly over independent repetitions of the digestion experiments, proving the reproducibility of the experimental set-up and digestion evaluations.

	C_f (%)	k (/min)
**CL30**	97.6 ± 2.1[a]	0.0258 ± 0.0015[a]
**$CL30_{rep1}$**	92.9 ± 3.4[a]	0.0329 ± 0.0033[a]
**$CL30_{rep2}$**	100.3 ± 2.6[a]	0.0302 ± 0.0018[a]

CL: Mechanically disintegrated whole lentil seeds cooked for 30 minutes;

rep: Repetitions of the whole experimental approach. Lentils of the same batch of raw material were cooked and mechanically disintegrated independently. Digestion experiments were carried out separately, using separately characterized enzyme batches.
